# [Retracted] miR‑143‑5p suppresses breast cancer progression by targeting the HIF‑1α‑related GLUT1 pathway

**DOI:** 10.3892/ol.2024.14444

**Published:** 2024-05-13

**Authors:** Jia Xu, Xi Li, Purong Zhang, Jie Luo, Exian Mou, Shiwei Liu

Oncol Lett 23: 147, 2022; DOI: 10.3892/ol.2022.13268

Following the publication of this article, a concerned reader has drawn to the attention of the Editorial Office that certain of the colony-formation assay data shown in Fig. 6F on p. 9 had already been published in the following paper, written by different authors at different research institutes: Wu X, Cui F, Chen Y, Zhu Y and Liu F: Long non-coding RNA LOXL1-AS1 enhances colorectal cancer proliferation, migration and nvasion through miR-708-5p/CD44-EGFR axis. Onco Targets Ther 13: 7615–7627, 2020.

Owing to the fact that the contentious data in Fig. 6F of the above article had already been published prior to its submission to *Oncology Letters*, the Editor has decided that this paper should be retracted from the Journal. The authors were asked for an explanation to account for these concerns, but the Editorial Office did not receive a reply. The Editor apologizes to the readership for any inconvenience caused.

